# Citrin deficiency due to SLC25A13 exon deletion in a Chinese infant: A case report

**DOI:** 10.1097/MD.0000000000036293

**Published:** 2023-12-08

**Authors:** Jialing Liu, Shuangzhu Lin, Shihui Guan, Qiandui Chen, Xinyao Wang, Yufei He, Yangfan Qi, Jinhua Feng, Yushu Liu

**Affiliations:** a Liuhe County Central Hospital, Liuhe Tonghua, Jilin Province, China; b The Affiliated Hospital of Changchun University of Chinese Medicine, Changchun, Jilin Province, China; c Changchun University of Chinese Medicine, Changchun, Jilin Province, China.

**Keywords:** aspartate aminotransferase, case report, citrin deficiency

## Abstract

**Introduction:**

Citrin is a calcium-bound aspartate-glutamate carrier protein encoded by the gene SLC25A13, mutations of which can cause citrin deficiency, an autosomal recessive disorder. The manifestations of citrin deficiency include neonatal intrahepatic choledeposits caused by citrin deficiency (NICCD: OMIM#605814), intermediate growth disorders and dyslipidemia caused by citrin deficiency, and citrullinemia type II (OMIM#603471) in adults. NICCD is a classical metabolic disorder that causes cholestasis in newborns.

**Patient concern and clinical findings:**

Here, we present the case of a 2-month-old male patient treated in our hospital on March 20, 2023, due to “postnatal skin xanthochromia and transaminases higher than normal values”. Since birth, the child’s skin had yellowed all over the body, and his condition did not improve after multiple medical treatments.

**Diagnosis/Intervention/Outcomes:**

The child underwent full exome gene testing at the age of 2 months and 13 days, and the results indicated heterozygous deletion of exon 3 of the SLC25A13 gene, while genetic testing of the parents revealed no gene mutations. The variant was preliminarily judged as being pathogenic according to the ACMG guidelines, and the patient was diagnosed with “citrin deficiency”. Skin yellowing eventually subsided, and liver function returned to normal without special treatment.

**Conclusions:**

Here, we report a rare case of citrin deficiency caused by a heterozygous deletion of the SLC25A13 gene. This case increases the clinical phenotypic profile of NICCD, suggesting that clinicians must be vigilant regarding such genetic metabolic diseases in the clinic for early diagnosis and treatment. NICCD should always be considered in the differential diagnosis of neonatal cholestasis.

## 1. Introduction

Citrin is a calcium-bound aspartate-glutamate carrier protein encoded by the SLC25A13 gene, located on chromosome 7q21.3.^[1–3]^ The citrin protein consists of 675 amino acids and has a molecular weight of 74 kDa. It is localized to the inner mitochondrial membrane and has 6 transmembrane spans, while it is expressed in the liver, small intestine, kidney, and heart.^[4]^ The highest expression is found in the liver.

Citrin deficiency is an autosomal recessive disorder caused by mutations in the SLC25A13 gene. Citrin deficiency includes a wide range of clinical phenotypes, including neonatal intrahepatic choledeposits caused by citrin deficiency (NICCD: OMIM#605814),^[2]^ intermediate growth disorders, and dyslipidemia caused by citrin deficiency,^[5]^ and citrullinemia type II (OMIM#603471) in adults.^[6]^ NICCD is a classic metabolic disorder that causes neonatal cholestasis,^[7]^ generally occurring between birth and 6 months, and presenting as mild aspartate aminotransferase (AST)/alanine aminotransferase (ALT) elevation, high direct bilirubin levels, hypoglycemia, and abnormal coagulation test results. This condition is also associated with high levels of galactose, citrulline, arginine, threonine/serine ratio, and alpha-fetoprotein.^[8]^ In 2001, Ohura et al^[9]^ were the first to identify a mutation in the SLC25A13 gene as causative of NICCD.

## 2. Case presentation

The patient was a male infant aged 2 months who was initially treated in our hospital on March 20, 2023, due to “postnatal skin xanthochromia and transaminases higher than normal values”. The child was born on January 24, 2023, with a length of 49 cm (average) and a weight of 2.9 kg (average) at birth. He was admitted to the local hospital for treatment due to yellow skin; physical and chemical examination revealed higher than normal total bile acid, ALT, and AST values. Light therapy was subsequently performed; however, the patient was discharged without improvement. At the age of 2 months, the child was 51.5 cm long (−2SD), weighed 3.8 kg (−2SD), whole body skin yellowing did not improve, and was accompanied by growth retardation. Following admission to our hospital, liver function tests revealed that the ALT was 96 U/L (reference value < 50 U/L) and AST was 69 U/L (reference value < 40 U/L). As both indicators were higher than normal, we administered “compound glycyrrhizin tablets” orally for 7 days; however, yellow skin staining remained. Repeated liver function showed that AST returned to normal, and ALT was 107U/L, as such, we administered “glutathione” pump therapy for 5 days, and the child’s ALT rose to 114 U/L. The patient was further treated at Beijing Children’s Hospital, where he underwent whole exome gene testing at the age of 2 months and 13 days and was diagnosed with “citrin deficiency” without undergoing special treatment. On August 22, 2023, when the child was 6 months and 12 days old, he was admitted to our hospital again. The yellow staining of the child’s skin had subsided, and the parents informed us that the liver function review had returned to normal, but growth retardation was still present. At this time, the child was 64 cm long (−2SD) and weighed 7 kg (−2SD); the parents further informed us that the child was 55 cm long (−2SD) and weighed 4.4 kg (−2SD) at 4 months of age.

The child was born G1P1 and delivered spontaneously at 39 + 5 weeks of gestation. He had no history of asphyxia or hypoxia, the mother showed no special history during pregnancy, with no prior diseases, and no similar family history. The patient was mixed-fed after birth, with deep hydrolyzed milk powder changed to ordinary milk powder for mixed feeding after 4 months. The child presented with motor development delay and could only raise his head at 3 months of age, could lie on his side at 4 months of age, and at 6 months of age, could turn over but could not sit up unassisted.

### 2.1. Physical examination

The child was 2 months and 6 days old at the time of admission, with a length of 51.5 cm (−2SD), a weight of 3.8 kg (−2SD), clear consciousness, moderate nutrition, yellow staining of the skin, mild yellow stain of the sclera, and non-palpable superficial lymph nodes. Listen and chase were normal, and he could erect his head briefly. The conjunctiva of both eyes was not hyperemeal, the cornea was transparent, the bilateral pupil and other large circles were sensitive to light reflection, and the chasing light was normal. He showed normal hearing, no cyanosis of the lips, no abnormalities in the jaw arch, the tongue protruding into the center, and his neck was soft. Breathing sounds were clear in both lungs, with no wet or dry rales. He had a heart rate of 130 beats/minutes, with no obvious abnormal murmur heard in the auscultation area of each valve. The abdomen was soft, and the liver and spleen were not palpable under the ribs. No abnormalities were observed in the spinal extremities, anus, or external genitalia. Limb muscle tone and muscle strength were normal, physiological reflexes were present, and pathological reflexes were not elicited.

### 2.2. Laboratory and imaging results

Routine blood test results were as follows: white blood cells: 9.95 × 10^9^/L, red blood cells: 3.75 × 10^12^/L, platelets: 374 × 10^9^/L, lymphocyte percentage: 78.7%, neutrophil percentage: 8.4%, no abnormalities; 1.25-hydroxyvitamin D: 42.65 ng/mL. Trace elements: calcium: 2.64 mmol/L, phosphorus: 1.97 mmol/L, no abnormalities were observed; Hepatitis B quantitative, infectious pediatrics (hepatitis A, hepatitis B, hepatitis C, hepatitis E, syphilis), respiratory syncytial virus, Epstein-Barr virus, Touch virus were not abnormal.

Liver function tests were performed from birth to 4 months of age, presented in Table 1.

**Table 1:** Results of liver function analyses over time.

Date:	January 26, 2023	January 30, 2023	March 26, 2023	April 02, 2023	April 08, 2023
ALT	105 U/L	99 U/L	96 U/L	107 U/L	114 U/L
AST	94 U/L	88 U/L	69 U/L	37 U/L	33 U/L

ALT = alanine aminotransferase, AST = aspartate aminotransferase.

### 2.3. Imaging examination

Liver and spleen ultrasound and electrocardiography were performed at 10 days, 2 months, and 4 months of age, revealing no abnormalities.

### 2.4. Further examination

**Figure 1.**
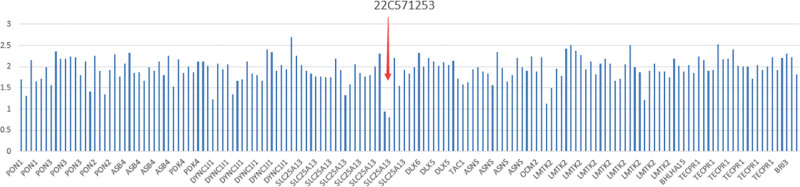
Results of genetic testing.

When the child was treated in Beijing Children’s Hospital, due to continuous yellowing of the skin after birth, transaminases continued to be higher than normal values, there was slow growth and development, and genetic metabolic diseases could not be ruled out. Therefore, after obtaining informed consent from the parents on April 6, 2023, whole exome gene testing was performed for the child and his parents. The results indicated heterozygous deletion of exon 3 of the SLC25A13 gene (Fig. 1), which was preliminarily determined to be a pathogenic variant according to ACMG guidelines. Genetic testing of the parents revealed no mutations.

## 3. Discussion

The human SLC25A13 gene encodes hepatic aspartate/glutamate carrier isoform 2 protein, more commonly known as citrin.^[10,11]^ This protein facilitates the exchange of mitochondrial aspartate with cytosolic glutamic acid and thus plays an important role in the urea, protein, and nucleotide synthesis pathways. Thus, under conditions of citrin deficiency, mitochondria are unable to transport aspartate into the cytoplasm, resulting in defects in arginine succinate synthesis, citrullinemia, and hyperammonemia. Loss of limonin impairs urea and protein synthesis, aerobic glycolysis, glycogen production, and energy metabolism.^[4]^

NICCD, a citrin deficiency disorder, is characterized by transient intrahepatic cholestasis of a variety of amino acids, including citrulline, methionine, arginine, threonine, phenylalanine, and tyrosine. Other manifestations include hypergalactosemia, elevated serum total bile acids, alpha-fetoprotein, and pancreatic secretory trypsin inhibitors, low birth weight, hypoproteinemia, hypoglycemia, decreased clotting factors, and failure to thrive.^[4]^ While these symptoms usually resolve by 1 year of age, only a small number of patients progress to liver failure requiring liver transplantation, and some infants develop citrullinemia type II decades later.^[12]^ At the same time, many patients with NICCD are asymptomatic in infancy.^[13]^ Liver histopathology for NICCD generally shows fatty liver, steatohepatitis, liver fibrosis, and rarely cirrhosis. Cholestasis and fatty liver usually improve by 1 year of age.^[14]^

Our child showed skin yellowing after birth, and chemical examinations showed that the ALT and AST levels were higher than normal. The preliminary diagnosis was neonatal jaundice; however, the child’s condition did not improve after phototherapy, and he continued to show persistent skin yellowing and abnormal liver function. The subsequent digestive ultrasound did not identify any abnormalities, and we excluded jaundice, viral hepatitis, and other diseases, as well as liver damage caused by hepatotropic virus infection including cytomegalovirus, Epstein–Barr virus, Torch virus, etc. The patient also had no history of taking drugs that may cause liver damage after birth, and the possibility of drug-induced liver damage was therefore also ruled out. After 7 days of oral compound glycyrrhizin tablets, AST returned to normal, but ALT did not decrease significantly, so we administered glutathione pump therapy for 5 days, and subsequent liver function review showed that ALT had increased again. To further clarify the diagnosis and treatment, the child was treated at Beijing Children’s Hospital. Considering the growth retardation after birth and the persistence of elevated ALT, the possibility of genetic metabolic diseases could not be ruled out, and the whole exon testing was performed on both the child and parents after obtaining informed consent. The test results indicated heterozygous deletion of exon 3 of the SLC25A13 gene, and the variant was preliminarily determined to be pathogenic according to the ACMG guidelines. The final diagnosis of NICCD was made.

At least 31 SLC25A13 gene mutations have been identified in patients with citrin deficiency in the literature, of which c.851_854del and c.615 + 5G NA mutations account for 80% of Chinese patients with citrin deficiency.^[4]^ Unlike the other reported cases, the present patient showed a persistent increase in ALT on physical and chemical examination, but this was not accompanied by typical high levels of galactose, citrulline, arginine, threonine/serine ratio, or alpha-fetoprotein, etc, and digestive ultrasound did not show any indications of fatty liver, steatohepatitis, liver fibrosis, or cirrhosis. Symptomatically, the present patient developed prolonged skin yellowing with growth retardation. This patient is the first reported case of citrin deficiency due to heterozygous deletion of pathogenic genes. Through follow-up visits, we learned that, as of now, the child is 6 months old, 64 cm (−2SD) long, and 7 kg (−2SD) in weight, has no yellow staining of the whole body, and liver function has returned to normal. We will continue to observe the child’s condition through follow-up visits and track any disease progression in the later stages of growth.

## 4. Conclusion

In conclusion, we report a rare case of citrin deficiency caused by a heterozygous deletion of the SLC25A13 gene. As the first reported case of this mutation, this report will increase the clinical phenotypic spectrum of NICCD, suggesting the need to be vigilant for such genetic metabolic diseases, to allow for early diagnosis and treatment. Simultaneously, we postulate that NICCD should always be considered in the differential diagnosis of neonatal cholestasis. Finally, the amino acid profile and high galactose levels detected in genetic and neonatal screening tests are important for the diagnosis of NICCD.

## Acknowledgements

We thank the patient and their family for their participation in this study.

## Author contributions

**Investigation:** Shuangzhu Lin, Shihui Guan.

**Resources:** Jialing Liu, Shuangzhu Lin, Shihui Guan, Yushu Liu.

**Software:** Yufei He.

**Supervision:** Shuangzhu Lin, Jinhua Feng, Yushu Liu.

**Writing – original draft:** Qiandui Chen, Xinyao Wang, Yufei He, Yangfan Qi.

**Writing – review & editing:** Qiandui Chen, Yangfan Qi, Jinhua Feng.
